# Novel Support Routing Algorithm for Quantum Satellite Networks with Finite Quantum Memory

**DOI:** 10.3390/e28070805

**Published:** 2026-07-15

**Authors:** András Mihály, László Bacsárdi

**Affiliations:** Department of Networked Systems and Services, Faculty of Electrical Engineering and Informatics, Budapest University of Technology and Economics, Műegyetem rkp. 3., H-1111 Budapest, Hungary; bacsardi@hit.bme.hu

**Keywords:** entanglement swapping, quantum network routing, quantum communication, quantum satellite network, line-graph, quantum memory saturation

## Abstract

Quantum memories are a critical component of entanglement-based quantum networks, enabling the storage and synchronisation of quantum states across dynamic links. However, current quantum memories have significantly lower capacity than the rate at which entanglement can be generated, making memory saturation a key bottleneck that reduces network efficiency and hinders the scaling of quantum networks. This problem is especially pronounced in dynamic satellite-based quantum networks, where short visibility windows constrain link availability. In this paper, we present a support entanglement-swapping algorithm that utilises leftover entanglement in quantum memories, thereby alleviating memory saturation and increasing network connectivity. Our algorithm combines two mathematical concepts, line graphs and maximum-cardinality matching, to select independent entanglement swap pairs without sharing any entanglement between concurrent swaps. This property ensures that the resulting changes to the network remain local and mutually independent, making the algorithm easy to integrate alongside any existing routing schemes without requiring network-wide coordination. We evaluate the algorithm through simulations on both static fibre-based networks and dynamic satellite networks. Across most configurations, our algorithm increases both the mean and the total number of entanglements shared between end nodes, while also increasing the network’s long-range connectivity. The ‘SwapWithToUse’ algorithm variant consistently provides the greatest improvements, with gains increasing as entanglement-generation rate increases.

## 1. Introduction

As the era of advanced quantum computers draws closer, the importance of the field of quantum communications grows twofold. Quantum communication can mitigate the risk posed by Shor’s algorithm [1] to the current public-key-based cryptographic systems through secure quantum-based solutions such as quantum key distribution (QKD). QKD allows us to generate symmetric keys while simultaneously detecting eavesdroppers securely [2]. Second, while advanced quantum computers will be able to run many quantum algorithms locally, which will play a key role in many fields, such as in healthcare [3], material engineering [4], and optimisation [5], the cost of building and running one can be astronomically high. By connecting quantum computers, we can create a network of quantum systems capable of running networked quantum services [6]. Two such key services are blind quantum computing [7] and distributed quantum computing [8]. Blind quantum computing enables running a quantum algorithm on a server without leaking any information about our algorithm or process, making it viable even for high-secrecy use cases. Distributed quantum computing, on the other hand, divides a larger quantum algorithm so that it can be run across multiple quantum systems.

Quantum communication provides inherent security guarantees rooted in fundamental quantum-mechanical principles, rather than relying on computational-complexity assumptions. Leveraging quantum phenomena such as the no-cloning theorem [9] ensures that these systems remain secure even against adversaries with quantum computational capabilities [10]. Quantum communication can further support secure information exchange through dedicated quantum key distribution networks [11]. Such networks differ from general quantum communication networks in that they typically exchange secrets rather than transmitting quantum states between parties. In this paper, we focus on the broader class of quantum communication networks intended for use as the quantum internet.

In most practical quantum communication implementations, photons act as the primary carriers of quantum information. The field can be divided into two channel types: fibre- and free-space-based. Fibre-based quantum networks operate over a controlled medium and benefit from compatibility with existing telecommunications infrastructure [12]. However, they are inherently limited in range and require fixed physical connections between nodes. Free-space quantum communication operates through uncontrolled atmospheric channels and enables the formation of dynamic links without requiring physical infrastructure between communicating parties. This property is especially valuable in satellite-based quantum networks, which can greatly extend coverage and connectivity [13] of the network. Consequently, a growing number of research efforts have explored the use of satellites for quantum key distribution (QKD) and the quantum internet [14]. A prominent example is the Micius satellite experiment [15], in which Chinese researchers demonstrated ground-to-satellite quantum state teleportation. Another important development by the researchers at the National University of Singapore [16] is an entanglement-based nanosatellite called SpooQy-1. On the European side, a more recent initiative involves the use of geostationary earth orbit (GEO) satellites for QKD [17], with Thales Alenia Space and Hispasat planning a secure key distribution network based on GEO infrastructure. Satellite-based quantum communication introduces distinct challenges in free-space links, where error rates vary as a function of both time and channel orientation [18]. The complexity of such systems differs considerably across orbital configurations. Low earth orbit (LEO) links benefit from shorter distances but are constrained by brief visibility windows. LEO-to-MEO (medium earth orbit) links involve longer propagation paths, which in turn increase error rates. LEO-to-GEO links, while offering extended connection durations, face the most severe distance-related challenges, requiring robust error correction to preserve quantum state fidelity over long distances.

Using satellites as intermediary nodes in a quantum network can offer numerous benefits, such as increased coverage or longer links. Still, it also introduces time dependence into the network. As satellites are in constant motion, even relative to each other, links appear and disappear over time. This dynamic nature of the network introduces a new problem: quantum memories of nodes that are not on any high-traffic route at the moment become saturated and later become unable to communicate efficiently. The entanglements in the quantum memories can still be utilised, so just throwing them away would be wasteful. In our research, we propose using leftover entanglements from routing to improve network performance and free up node quantum memories.

The structure of the paper is organised as follows. In Section 2, we provide additional background on dynamic quantum networks and quantum memory saturation. In Section 3, we present our novel algorithm, and in Section 4, we show the results obtained using it on both static and dynamic graphs. Finally, Section 5 concludes our paper.

## 2. Background

**Figure 1:**
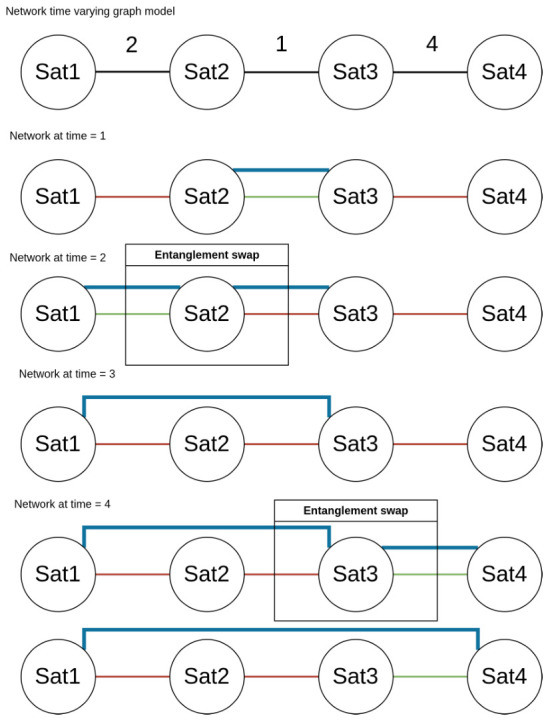
Example network for showing non-time linearity in quantum communication. Nodes represent communicating parties (Sat1: first satellite node, Sat2: second satellite node, and so on), and the edge weights in the first figure represent the time availability of the links. In subsequent figures, we visualise edge availability in green (available) or red (unavailable), and entanglements in blue.

Quantum memories are a key part of any future quantum entanglement-based network. Quantum memories enable the storage of quantum information over extended time, in some cases even up to 10 h [19]. Using these memories, we can create routes in dynamic quantum networks that would otherwise be infeasible. For example, let us take four satellite nodes named Sat1, Sat2, Sat3, and Sat4. In this example, we will use the visibility intervals shown in Figure 1. We can see in the figure that a classical route would not be possible between Sat1 and Sat4, but using quantum entanglement swapping [20,21], it is possible to create a pair of entangled quantum bits between the two. First, at t = 1, Sat2 and Sat3 create a pair of entangled quantum bits between them. Next, at t = 2, Sat2, after creating the entangled quantum bits with Sat1, applies quantum entanglement swapping, creating an entangled pair between Sat1 and Sat3. Lastly, at t = 4, Sat3 can repeat this process with Sat4, creating an entangled pair of quantum bits between Sat1 and Sat4. This process only requires quantum memories that can remain coherent for at least three time instances for our example.

Quantum memories today have a significantly lower capacity [22] in relation to the rate of quantum entanglement generation [23]. This inequality can lead to quantum memories reaching their capacity, becoming ‘saturated’. This quantum memory saturation, as we show later in Section 4, can prevent quantum memories from accepting new entanglements, thereby decreasing the network’s efficiency. In [24], researchers propose policies for the management of quantum memories of quantum switches. The proposed policy utilises memory for requests for entanglements between neighbouring nodes but does not address unused links in current requests, which can lead to memory saturation in dynamic networks.

In our work, we created an algorithm that utilises ‘leftover’ quantum entanglements in quantum memories to increase network connectivity. The algorithm can be easily combined with any routing algorithm, as it requires no setup or specific input.

## 3. Our Algorithm

In our research, we developed an algorithm that uses leftover entanglements in the network, thereby preventing saturation of quantum memories. This algorithm uses two existing mathematical concepts: line graphs [25] and maximum-cardinality matching [26]. By combining these two concepts, we created a novel algorithm that selects entanglement pairs for swapping, such that no entanglement is shared between the selected swaps. Performing entanglement swaps without coinciding entanglements can have multiple benefits: the changes in the network remain local, the resulting changes do not depend on other entanglement swaps, and, because the changes remain local, it is easier to implement different weighting algorithms.

**Figure 2:**
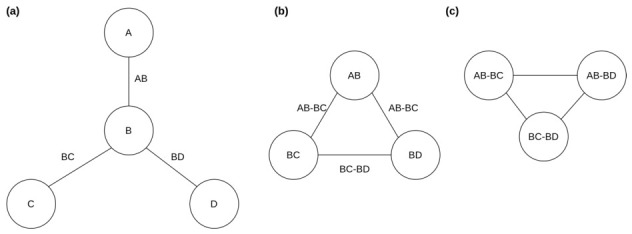
The process of constructing a line graph from an example graph with nodes labelled A, B, C, and D, and edges labelled by the concatenation of the node labels. (**a**) shows the original graph, (**b**) the first L1 line graph and (**c**) the final L2 line graph.

Our support entanglement swapping algorithm can be divided into two main steps: graph creation and matching. To create the graph, we take an undirected graph *G* of our network, where every node is in the ‘real’ network. Every two nodes are connected if there exists an entangled quantum bit pair between them. Next, we take the line graph of the network, resulting in $L_{1}=L\left(G\right)$. In $L_{1}$, by definition, every node is an edge in *G*, and there exists an edge between two nodes if the edges share a node in *G*. In $L_{1}$ every node can be thought of as an entanglement in the original network, and by creating the line graph of $L_{2}=L\left(L_{1}\right)$, as it can be seen in Figure 2, we get a graph where every node is an entanglement swap (a pair of entanglements) in the original network. All swaps that share an entanglement are neighbours. It is trivial to see that taking a maximal independent set in $L_{2}$ yields a list of entanglement swaps in the original network that do not share entanglements. In our work, we sought to develop an algorithm that leverages this property of $L_{2}$ graphs, since selecting entanglement swaps in this way yields newly generated independent entanglements without the possibility of reusing the same entanglement. This property can then be used to utilise ’unused’ entanglements in the network, freeing up quantum memories and increasing network connectivity, without wasting generated entanglements.

The main difficulty for this process is twofold: for arbitrary graphs, the size of their line graph grows with no bounds [27], and the problem of finding maximal independent sets is NP-complete [28]. This increase in complexity led to an algorithm in our research that was almost unusably slow. Minty [29] proved in 1980 that it is possible to find maximal sets in polynomial time for graphs that are line graphs ($L_{1}=L\left(G\right)$) of another graph by taking the maximal matching in the original graph (*G*). In our case, it means that finding a maximal matching in $L_{1}$ yields a maximal independent set in $L_{2}$.

In Algorithm 1, we describe our algorithm using pseudocode. The algorithm takes two main inputs: the network state representation *G* and the weight function $w_{f}$. In the first step, we calculate the $L_{1}$ line graph of *G*. Then, using our weight function, we calculate the $W_{E}$ weight of each edge in $L_{1}$. Next, we create the matching using $L_{1}$ and $W_{E}$. In the last step, we iterate over the elements of the matching, adding each as an edge pair to the selected_edges list. We later use this list in our simulator to perform the support entanglement swaps.
**Algorithm 1** Support routing algorithm**Input:** G=(V,E): graph representation of the network; $w_{f}$: weight function to be used**Output:** selected_edges: edge pairs selected by the algorithm  1:$L_{1}\leftarrow L\left(G\right)$                                                                   ▹ Calculated the line graph of G  2:$W_{E}\leftarrow w_{f}\left(e\right)$: $\forall \;e\in E_{L_{1}}$                                                                             ▹$L_{1}=\left(V_{L_{1}},E_{L_{1}}\right)$  3:match $\leftarrow \max\_\mathrm{weighted}\_\mathrm{matching}(L_{1},W_{E})$  4:**for** m∈ match **do**  5:      (edge_1,edge_2)=m  6:      selected_edges.append((edge_1,edge_2))  7:**end for**

**Figure 3:**
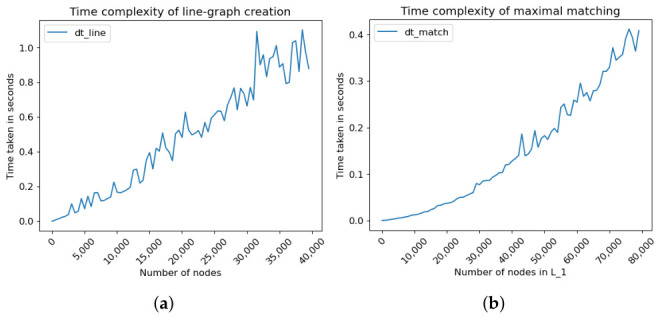
Simulated time complexity of the two key algorithms used in our algorithm. (**a**) shows how the time complexity of the line graph construction increases with the number of nodes in the original graph. (**b**) shows how the time complexity of the maximal matching increases with the number of nodes in the line graph $L_{1}$.

The complexity of our algorithm is the combination of the complexity of its two algorithms. The complexity of creating line graph $L_{1}=\left(V_{L},E_{L}\right)$ from G=(V,E) can be bounded by O(Δ(G)∗|V|) where Δ(G) is the maximal degree of G, while the complexity of maximal-cardinality matching is $O(|V_{L}{|}^{2}|E_{L}|)$ [26]. In Figure 3, we show the runtime of the key algorithms used in our algorithm. We used a connected Watts–Strogatz [30] graph with the following parameters: k=4, number of edges per new node; p=0.2, probability of edge rewiring; n∈[20,40,000]. In the figure, we can see that our algorithm scales well with larger graphs.

In addition to multiple types of networks, we also created three different versions of our algorithm, using various weighting functions for selecting the entanglement pairs to swap. The zeroth version of our algorithm is named ‘NoSwap’; in this scenario, we do not use the algorithm, only a simple round robin-style routing algorithm, where we iterate over all end node pairs, finding a single path between them using only one entanglement, repeating until no more paths can be found. The ‘SwapWithNoWeight’ scenario uses a simple graph representation of the network, where each edge indicates whether there is entanglement between the two nodes. In ‘SwapWithNewRoute’, we weighted each potential entanglement swap with the number of new routes it could create. In ‘SwapWithToUse’, we focused the weight function on finding a set of entanglement swaps that maximise the total number of used entanglements.

## 4. Results

In our research, we created a simulation framework to test and validate the usability of our algorithm. We used a deterministic simulator, meaning no randomness was introduced, allowing us to run each simulated network across our multiple parameter values only once. This results in around 100 simulations for each network algorithm combination. We simulated both static fibre networks and dynamic satellite networks. We included static networks for two key reasons. Static networks are easier to analyse thanks to their fixed architecture. This allows us to test the performance of our algorithm across different architectures, each with its unique features. The second reason for including static networks was to compare the effectiveness of our algorithm between static and dynamic networks. Our initial presumption was that using our algorithm would yield only marginal benefits in all cases, but, as we will show in this section, in some cases we could realise huge improvements across our test networks.

We tested our algorithms based on two main parameters: entanglement reservation and the entanglement percentage to use. Using the output of our algorithm, we need to apply entanglement swaps to the network, which naturally raises two key questions: how many entanglements to use and how many to leave? Entanglement reservation (*MaxEntToLeave*) allows us to control the amount of entanglements left after running the algorithm, while entanglement percentage (ToUsePercent) to use enables us to set the percentage of entanglements to use in the swaps. In Table 1, we present the main parameters used in our simulations, both for static and dynamic networks. We selected these values to showcase the performance of a network across various configurations. The values for memory size and entanglement generation were chosen so we can analyse networks where the memory saturates slowly and also when it saturates almost instantly. For the values related to our novel algorithm (MaxEntToLeave and ToUsePercent), we have chosen based on previous simulation runs.

### 4.1. Static Fibre Networks

**Figure 4:**
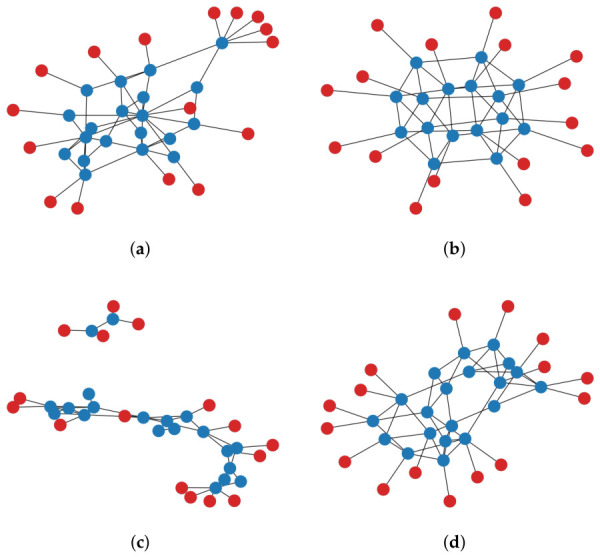
Network architectures used for simulation, end nodes shown in red, communication nodes shown in blue. (**a**) Barabási–Albert graph with parameters N=20 and M=2. (**b**) Hypercube graph with parameters $N=log_{2}20$. (**c**) random geometric graph with parameters N=20, r=0.3 and dim=2. (**d**) Connected Watts–Strogatz graph with parameters N=20, k=4 and p=0.2.

We created four distinct static architectures to test the effectiveness of our algorithm. A visual description of all four static architectures can be seen in Figure 4. We ran the three alternative algorithms for each static architecture and parameter combination with a simulation time of 1000 s. As these networks were imaginary, we assigned each connection a transmittance of 0.1, meaning that out of 1000 shared quantum bits, only 100 would arrive. Using this setup, we simulated and compared our algorithm across multiple types of network architectures.

#### 4.1.1. Barabási–Albert Graph

The Barabási–Albert [31] (BaAl) model generates a random scale-free network by starting with a small initial graph and iteratively adding new nodes, each connecting to *m* existing nodes. New nodes attach preferentially—nodes with higher degree are more likely to receive new edges, producing a “rich get richer” dynamic that results in a power-law degree distribution.

**Figure 5:**
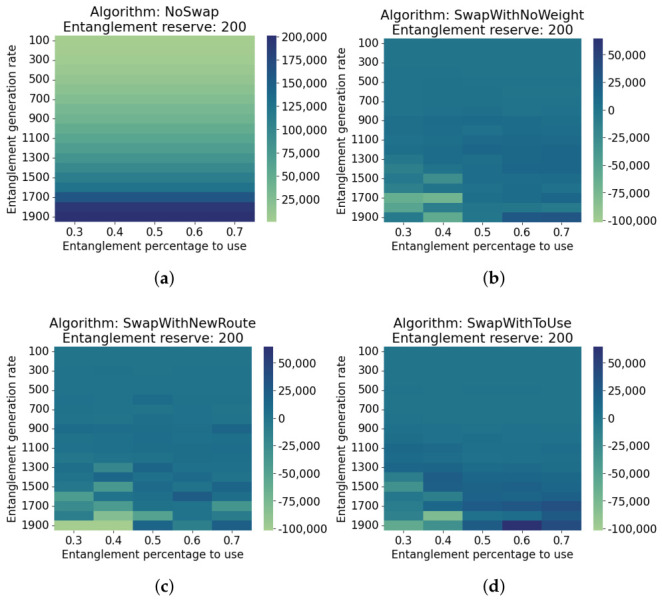
Results for the Barabási–Albert graph, showing the difference in mean entanglements shared between end nodes between ‘NoSwap’ and the different algorithms used. (**a**) Entanglements are shared between end nodes, using the ‘NoSwap’ algorithm. (**b**) Difference between ‘NoSwap’ and ‘SwapWithNoWeight’. (**c**) Difference between ‘NoSwap’ and ‘SwapWithNewRoute’. (**d**) Difference between ‘NoSwap’ and ‘SwapWithToUse’.

In Figure 5, we compare the three algorithm variants with the ‘NoSwap’ case. In Figure 5a we show the mean of shared entanglements between end nodes for each simulation configuration, then in Figure 5b–d we highlight the differences for each configuration with the use of our algorithms. We can see that, with high entanglement usage, some algorithms increase the mean of shared entanglements. We can also see in Figure 5d that the largest increase is achieved with the ‘SwapWithToUse’ algorithm, which prioritises selecting edge pairs to swap with the highest amount of entanglements.

**Figure 6:**
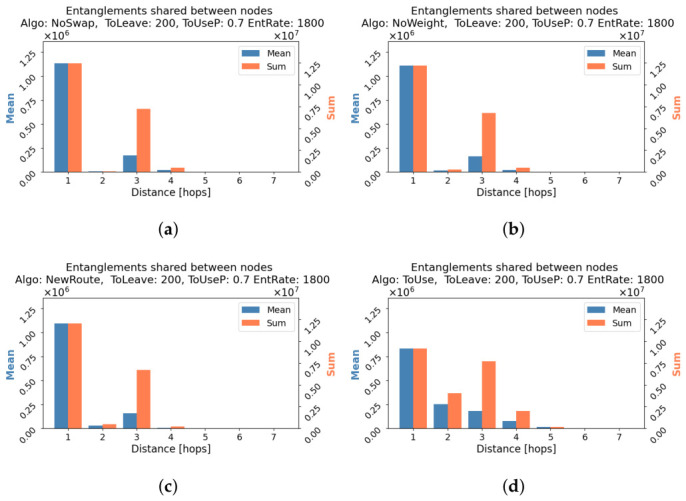
Bar chart showing the mean and total entanglements shared, grouped by the number of hops between the end nodes, for the Barabási–Albert graph. Entanglements are shared between end nodes, using the ‘NoSwap’ (**a**), ‘SwapWithNoWeight’ (**b**), ‘SwapWithNewRoute’ (**c**), and ‘SwapWithToUse’ (**d**) algorithms.

In Figure 6, we compare the four cases for a single configuration, with each sub-figure showing the mean and total generated entanglements across different distances. We can see that the ‘SwapWithToUse’ algorithm not only has the highest mean and total shared entanglements, but also connects end nodes that are the farthest apart.

#### 4.1.2. Hypercube Graph

A Hypercube graph (HYPER) is a deterministic graph where each node represents an n-bit binary string and two nodes are connected if and only if their strings differ by exactly one bit, yielding $2^{n}$ nodes each with degree *n*. It has a highly regular structure, low diameter (*n*), and is widely used to model parallel computing interconnects.

**Figure 7:**
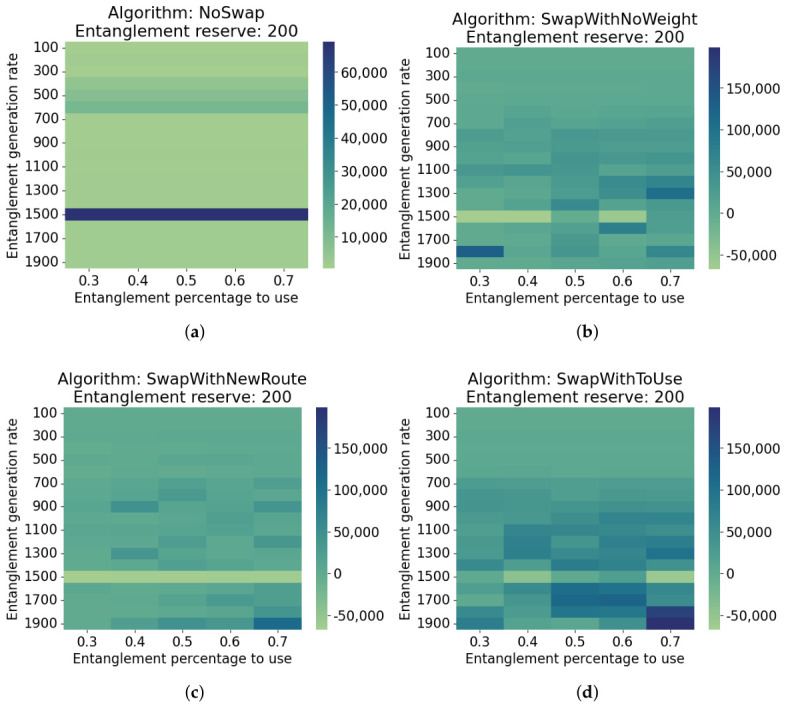
Results for the Hypercube graph, showing the difference in mean entanglements shared between end nodes between ‘NoSwap’ and the different algorithms used. (**a**) Entanglements shared between end nodes, using ‘NoSwap’ algorithm. (**b**) Difference between ‘NoSwap’ and ‘SwapWithNoWeight’. (**c**) Difference between ‘NoSwap’ and ‘SwapWithNewRoute’. (**d**) Difference between ‘NoSwap’ and ‘SwapWithToUse’.

In Figure 7, we can see the same pattern as before in the Barabási–Albert graph. The ‘SwapWithToUse’ algorithm provides the highest increase among all the others, and as entanglement usage percentages increase, the difference widens. It is interesting to note the dark blue line in Figure 7a, which shows an enormous increase in the mean of shared entanglements between end nodes.

**Figure 8:**
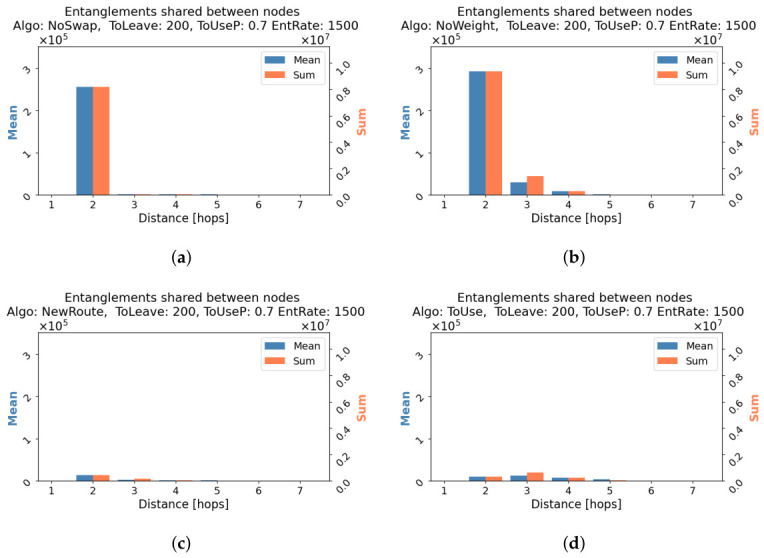
Bar chart showing the mean and total entanglements shared, grouped by the number of hops between the end nodes, for the Hypercube graph. Entanglements are shared between end nodes, using the ‘NoSwap’ (**a**), ‘SwapWithNoWeight’ (**b**), ‘SwapWithNewRoute’ (**c**), and ‘SwapWithToUse’ (**d**) algorithms.

As shown in Figure 8, this is because our exemplary round robin routing finds mostly shorter paths in the network, which is most likely due to network clogging caused by memory saturation in some specific way due to increased entanglement generation. We can also see that, in this case, one algorithm (‘SwapWithNoWeight’ Figure 7b) still led to an improvement in the network. The figure also shows the increased connectivity provided by our algorithm: as seen in Figure 8b, the ‘SwapWithNoWeight’ algorithm provides the best mean and total shared entanglement between end nodes while also increasing connectivity.

#### 4.1.3. Random Geometric Graph

In random geometric graphs [32] (RGEO), nodes are placed uniformly at random in a unit square (or d-dimensional space), and two nodes are connected if their Euclidean distance falls below a threshold radius *r*. This produces spatially clustered connectivity that naturally models wireless sensor networks and other proximity-based systems.

**Figure 9:**
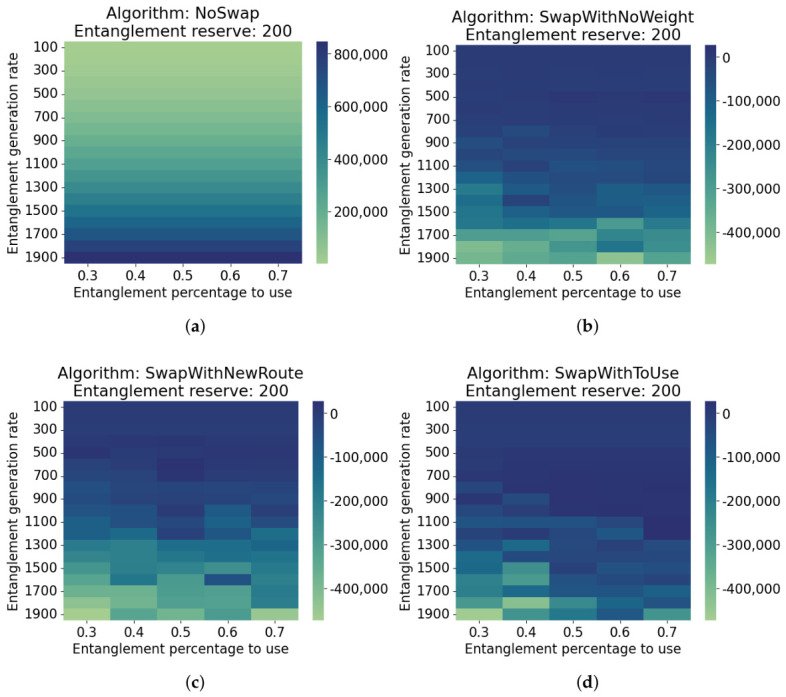
Results for the random geometric graph, showing the difference in mean entanglements shared between end nodes between ‘NoSwap’ and the different algorithms used. (**a**) Entanglements shared between end nodes, using ‘NoSwap’ algorithm. (**b**) Difference between ‘NoSwap’ and ‘SwapWithNoWeight’. (**c**) Difference between ‘NoSwap’ and ‘SwapWithNewRoute’. (**d**) Difference between ‘NoSwap’ and ‘SwapWithToUse’.

In Figure 9, we can see that the application of our algorithms does not lead to any increase in the mean of shared entanglements; it even decreases it for higher entanglement generation rates! This is due to the network being disconnected and having lower interconnectedness, as visible in Figure 4c.

#### 4.1.4. Connected Watts–Strogatz Graph

Connected Watts–Strogatz [30] (WaSt) is built by starting with a regular ring lattice where each node connects to its k nearest neighbours, then rewiring each edge independently with probability *p* to a random node, but constrained so that the resulting graph remains connected. The result interpolates between a regular lattice (p=0) and a random graph (p=1), exhibiting the small-world property of high clustering combined with short average path lengths for intermediate values of *p*.

**Figure 10:**
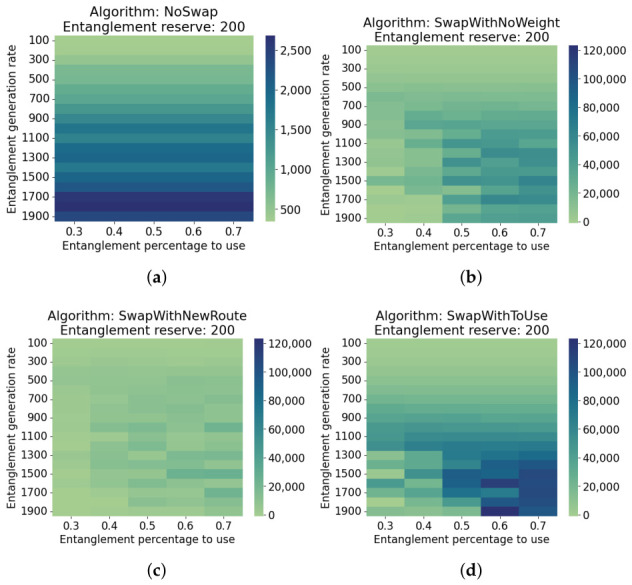
Results for the Connected Watts–Strogatz graph, showing the difference in mean entanglements shared between end nodes between ‘NoSwap’ and the different algorithms used. (**a**) Entanglements shared between end nodes, using ‘NoSwap’ algorithm. (**b**) Difference between ‘NoSwap’ and ‘SwapWithNoWeight’. (**c**) Difference between ‘NoSwap’ and ‘SwapWithNewRoute’. (**d**) Difference between ‘NoSwap’ and ‘SwapWithToUse’.

In Figure 10, we can see that with high entanglement usage percentages, all algorithms provide an increase in the mean of shared entanglements. We can also see in (**d**) that the largest increase is achieved with the ‘SwapWithToUse’ algorithm, which prioritises selecting edge pairs to swap with the highest amount of entanglements.

In Table 2, we show the improvement ratio for the different network architectures. The ratios were calculated by dividing the total/mean shared entanglements between end nodes for each algorithm by the total/mean shared entanglements by the ‘NoSwap’ algorithm. The table shows that the ‘SwapWithToUse’ algorithm consistently achieves the highest performance gains across all architectures, while the ‘SwapWithNewRoute’ algorithm generally provides the lowest increase among the three variants. The most pronounced improvements can be observed in the Watts–Strogatz and Hypercube architectures, where ‘SwapWithToUse’ achieves total ratios of 23.07 and 14.74, respectively, with even larger gains in the mean ratio: 42.90 for Watts–Strogatz and 54.06 for Hypercube. This suggests that in well-connected, regular network topologies, the algorithm is particularly effective at redistributing leftover entanglement along paths left by the primary round robin routing algorithm.

The Barabási–Albert graph shows more modest but consistent gains, with ‘SwapWithToUse’ achieving a total ratio of 1.72 and a mean ratio of 1.14. The scale-free nature of this topology, in which a small number of high-degree hub nodes dominate connectivity, likely limits the number of independent swap pairs the algorithm can identify, thereby reducing the overall benefit. In the random geometric graph, all three algorithm variants provide little to no improvement, with mean ratios falling below 1.0 for ‘SwapWithNoWeight’ and ‘SwapWithNewRoute’. This aligns with the disconnected, spatially clustered structure of this topology shown in Figure 4c: in a poorly interconnected network, entanglements consumed by the swap algorithm are no longer available for direct routing requests, meaning the algorithm competes with rather than complements the base routing scheme.

In Table 3, we compare the performance of our algorithms by calculating for each algorithm the correlation between the added benefit and the entanglement generation rate. We calculate the added benefit of an algorithm by taking the total entanglements shared in the network when using the respective algorithm, and subtracting the entanglements shared when using no supplementary algorithm (NoSwap). We then calculated the Pearson correlation for each algorithm at varying entanglement generation rates. We can see in Table 3 that for each network architecture, the algorithm with the highest correlation value is ‘SwapWithToUse’. This result coincides with our previous observations that, for most architectures, the ‘SwapWithToUse’ algorithm is the best choice.

Lastly, we show that increasing the number of entanglements used by our algorithms can lead to greater improvement. In Table 4, we show the correlation between the correlation level calculated for the previous Table 3 and the percentage of entanglements used by the supplementary algorithm. We show that, in almost all cases, there is a positive correlation between the value of the entanglement percentage to use and the correlation between the benefits and the entanglement generation rates. We can further see that the ‘SwapWithToUse’ algorithm has the highest overall correlation for each architecture, further proving that this algorithm has the highest potential of those compared.

### 4.2. Dynamic Satellite Networks

In our work, we used multiple satellite constellations, with different architectures (Cross and Retro) and numbers of satellites, ranging from 16 to 64. The two main architectures are used to create two distinct dynamic networks. In the Cross constellation, every second satellite’s inclination is shifted by 90°, creating an interconnected cross-like shape. Retro constellations shift the satellite’s inclination by 180°. This leads to networks with longer visibility intervals for Cross constellations and shorter ones for Retro constellations. This difference between the two constellations allowed us to analyse different dynamic networks. For the base for satellite orbits, we used a Starlink satellite orbit and modified it to create the constellations. A more detailed description of the satellite orbits in the constellation is shown in Table 5.

In our research, we calculated satellite link losses between satellites using the QSCS 2.0 [33] developed at our university. The tool allowed us to specify default parameters for the whole simulation, along with key parameters for loss calculations. The full list of parameters is shown in Table 6, including both dynamic and static parameters.

In our work, we found that increasing the number of satellites in the constellation does not yield patterns that differ significantly from those of the original LOW constellations. For this reason, we only show selected bar chart figures for constellations with a higher number of satellites.

#### 4.2.1. Cross Architecture

**Figure 11:**
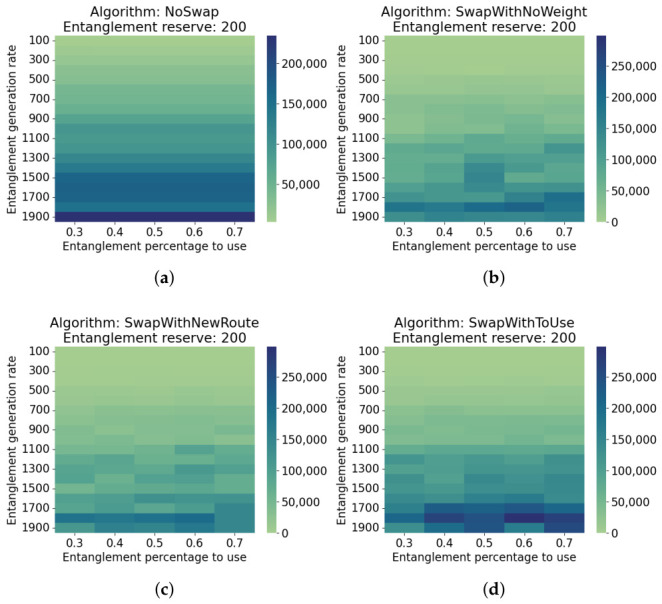
Results for the Cross LOW constellation, showing the difference in mean entanglements shared between end nodes between ‘NoSwap’ and the different algorithms used. (**a**) Entanglements shared between end nodes, using ‘NoSwap’ algorithm. (**b**) Difference between ‘NoSwap’ and ‘SwapWithNoWeight’. (**c**) Difference between ‘NoSwap’ and ‘SwapWithNewRoute’. (**d**) Difference between ‘NoSwap’ and ‘SwapWithToUse’.

In constellations using the Cross architecture, every second orbit’s inclination is shifted by 90°, resulting in longer visibility intervals. This setup leads to longer intersatellite visibility intervals, making the resulting network less time-dependent, allowing more time per edge. As shown in Figure 11, our proposed algorithms increase the average shared entanglement between end nodes across all cases, with higher entanglement generation rates leading to larger increases.

**Figure 12:**
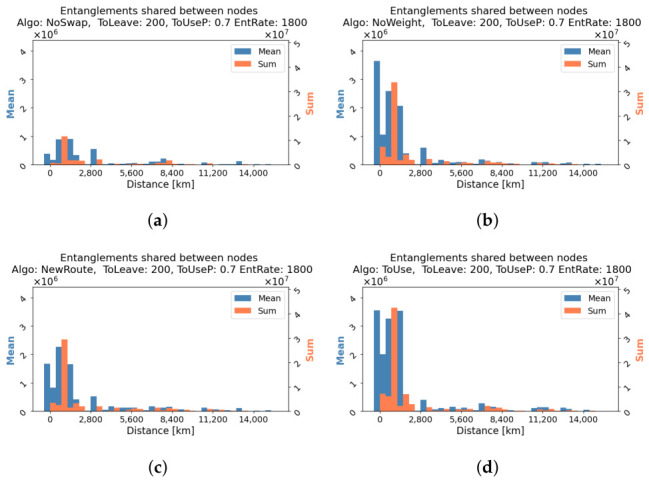
Bar chart of Cross LOW constellation showing the mean and total entanglements shared grouped by the number of hops between the end nodes. (**a**) Entanglements shared between end nodes, using ‘NoSwap’ algorithm. Difference between ‘NoSwap’ and ‘SwapWithNoWeight’ (**b**), ‘SwapWithNewRoute’ (**c**), and ‘SwapWithToUse’ (**d**).

**Figure 13:**
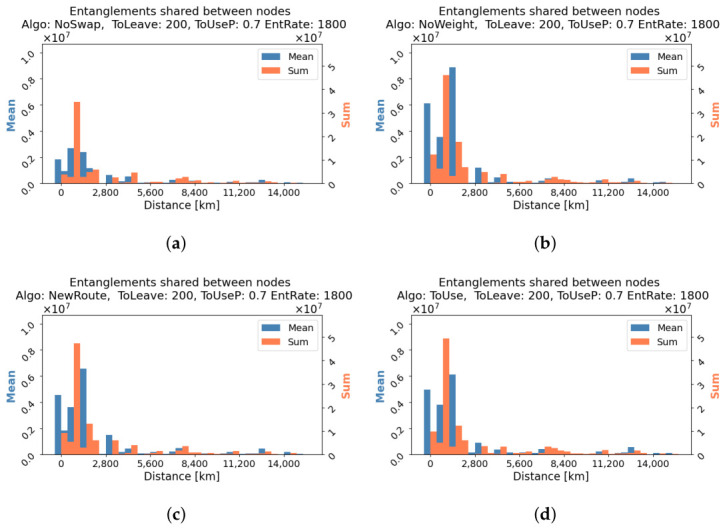
Bar chart of Cross LOWMID constellation showing the mean and total entanglements shared grouped by the number of hops between the end nodes. (**a**) Entanglements shared between end nodes, using ‘NoSwap’ algorithm. Difference between ‘NoSwap’ and ‘SwapWithNoWeight’ (**b**), ‘SwapWithNewRoute’ (**c**), and ‘SwapWithToUse’ (**d**).

In Figure 12, we can observe that, as in the case for static networks, our algorithms not only increased the average of shared entanglements but also the connectivity of the network, leading to entanglements shared across higher distances. We can also see in Figure 13d that the ‘SwapWithToUse’ algorithm provides the best increase in connectivity.

**Figure 14:**
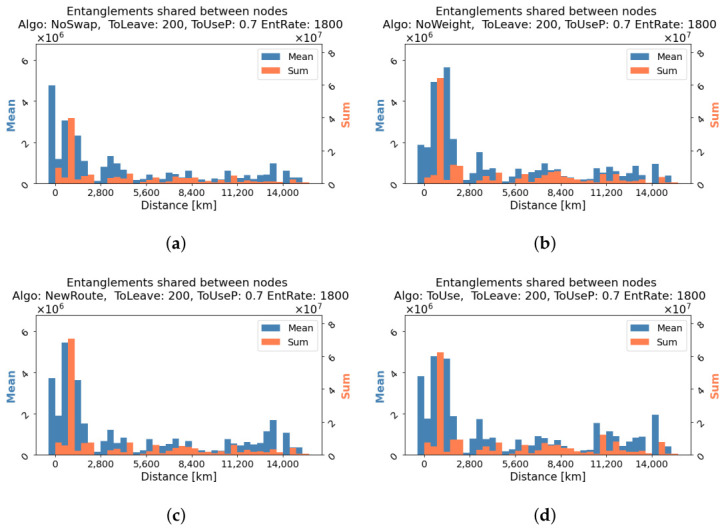
Bar chart of Cross MID constellation showing the mean and total entanglements shared grouped by the number of hops between the end nodes. (**a**) Entanglements shared between end nodes, using ‘NoSwap’ algorithm. Difference between ‘NoSwap’ and ‘SwapWithNoWeight’ (**b**), ‘SwapWithNewRoute’ (**c**), and ‘SwapWithToUse’ (**d**).

As we can see in Figure 13 and Figure 14, at a higher number of satellites, our algorithms still provide a high level of improvement in both distance and throughput.

As shown in Table 7, the Cross constellation architectures follow the same pattern observed in static networks: ‘SwapWithToUse’ consistently achieves the highest gains in both total and mean entanglement shared between end nodes, with ‘SwapWithNewRoute’ providing the most modest improvements across all constellation sizes. The table also shows that across all constellations, the ‘SwapWithToUse’ algorithm provides the greatest performance increase. The Cross LOW constellation achieves the highest gains, with ‘SwapWithToUse’ reaching a total ratio of 2.4 and a mean ratio of 2.0, while Cross MID yields ratios of 1.49 and 1.54, respectively. This is likely because larger constellations can provide higher throughput, which cannot be increased indefinitely due to the bottlenecking of low-throughput Ground-Satellite channels.

Beyond the improvement in raw entanglement counts, our algorithm also increases the long-range connectivity of the network, as shown in Figure 14: end nodes separated by larger distances, which share no entanglement under the ‘NoSwap’ baseline, can communicate when any of the three algorithm variants is applied. This connectivity gain is most pronounced for the ‘SwapWithToUse’ variant, and represents a qualitative improvement in network capability beyond what the ratio values alone capture.

#### 4.2.2. Retro Architecture

**Figure 15:**
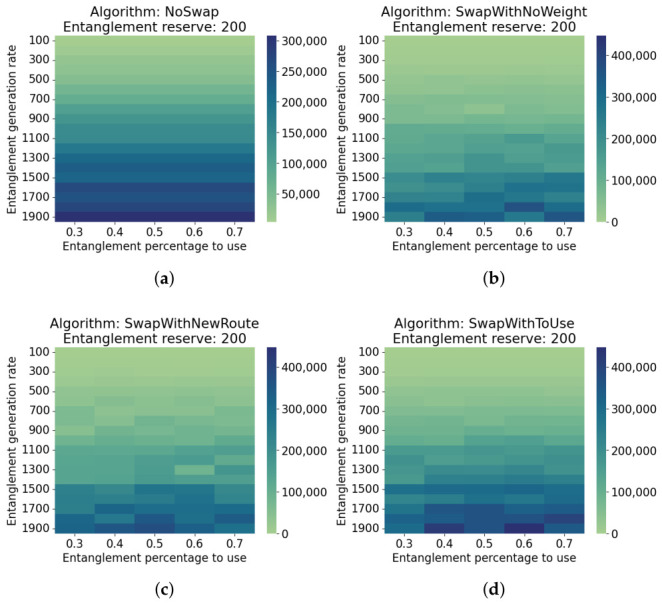
Results for the Retro LOW constellation, showing the difference in mean entanglements shared between end nodes between ‘NoSwap’ and the different algorithms used. (**a**) Entanglements are shared between end nodes, using the ‘NoSwap’ algorithm. Difference between ‘NoSwap’ and ‘SwapWithNoWeight’ (**b**), ‘SwapWithNewRoute’ (**c**), and ‘SwapWithToUse’ (**d**).

In constellations using the Retro architecture, every second orbit’s inclination is shifted by 180°, resulting in shorter visibility intervals. This leads to shorter inter-satellite visibility intervals, making the resulting network more time-dependent. As shown in Figure 15, as in the case of the Cross architecture, our proposed algorithms increase the average shared entanglements between end nodes in almost all cases, with higher entanglement generation rates leading to larger increases.

**Figure 16:**
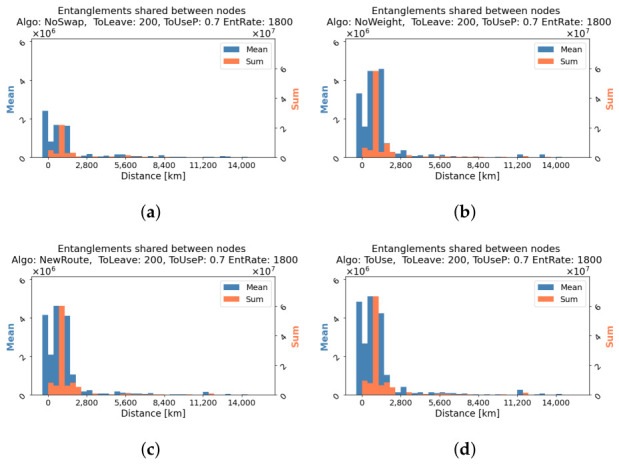
Bar chart showing the mean and total entanglements shared, grouped by the number of hops between the end nodes, for the Retro LOW constellation. Entanglements are shared between end nodes, using the ‘NoSwap’ (**a**), ‘SwapWithNoWeight’ (**b**), ‘SwapWithNewRoute’ (**c**), and ‘SwapWithToUse’ (**d**) algorithms.

In Figure 16, we can observe that, as in the case for static networks, our algorithms not only increased the average of shared entanglements but also the connectivity of the network, leading to entanglements shared across higher distances. We can also see in Figure 16d that the ‘SwapWithToUse’ algorithm provides the best increase in connectivity, although not at the same rate as for the Cross architecture, suggesting that the constellation’s architecture is related to the algorithm’s effectiveness.

**Figure 17:**
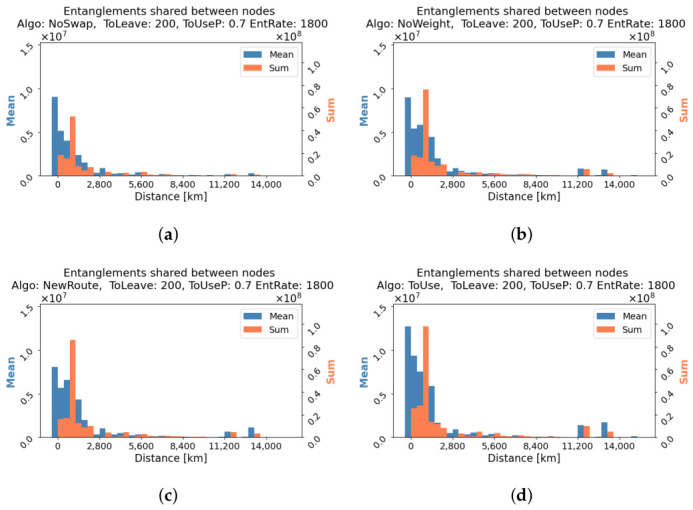
Bar chart of Retro LOWMID constellation showing the mean and total entanglements shared grouped by the number of hops between the end nodes. (**a**) Entanglements shared between end nodes, using ‘NoSwap’ algorithm. Difference between ‘NoSwap’ and ‘SwapWithNoWeight’ (**b**), ‘SwapWithNewRoute’ (**c**), and ‘SwapWithToUse’ (**d**).

**Figure 18:**
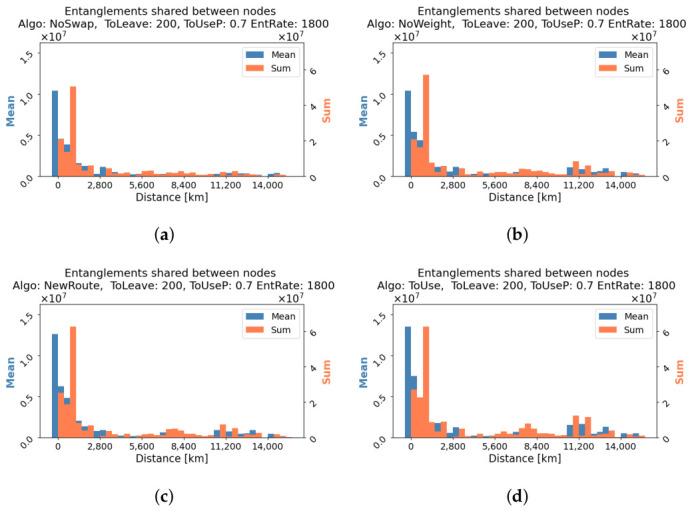
Bar chart of Retro MID constellation showing the mean and total entanglements shared grouped by the number of hops between the end nodes. (**a**) Entanglements shared between end nodes, using ‘NoSwap’ algorithm. Difference between ‘NoSwap’ and ‘SwapWithNoWeight’ (**b**), ‘SwapWithNewRoute’ (**c**), and ‘SwapWithToUse’ (**d**).

In Figure 17 and Figure 18, we show that increasing the number of satellites results in increased long-distance connectivity, like in the case of the Cross architecture.

We detail in Table 8 that the Retro constellation architectures follow the same general pattern as the Cross constellations: ‘SwapWithToUse’ consistently achieves the highest gains in both metrics across all constellation sizes, ‘SwapWithNewRoute’ provides the most modest improvements. All three variants yield substantial increases over the ‘NoSwap’ baseline, with no architecture showing degradation. When comparing the results directly with the Cross architecture results in Table 7, our algorithm consistently underperforms in total entanglement ratio. For example, ‘SwapWithToUse’ achieves a total ratio of 2.45 for Cross LOW compared to 2.17 for Retro LOW, with the gap closing in the mean ratio: 2.06 versus 2.14. This is likely due to shorter intersatellite links, resulting in a less connected network, and the Retro constellations already having a higher total of shared entanglement. In parallel with the Cross architecture case, our algorithm provides increased connectivity and connection rate for every constellation. For larger constellations, we can observe a similar decrease in the amount of entanglements shared between end nodes.

In Table 9, we compare the performance of our algorithms by calculating for each algorithm the correlation between the added benefit and the entanglement generation rate. We calculate the improvement of an algorithm by subtracting the entanglements shared when using the respective algorithm from those shared when using no supplementary algorithm (NoSwap). We then calculated the Pearson correlation for each algorithm at varying entanglement generation rates. The table shows that, for each network architecture, the algorithm with the highest correlation is ‘SwapWithToUse’. This result coincides with our previous observations for both static and dynamic networks that, for most architectures, the ‘SwapWithToUse’ algorithm is the best choice.

As with static networks, we show in Table 10 that the amount of improvement provided by our algorithm correlates with the amount of entanglements it consumes (**ToUsePercent** value). In the table, we showcase the correlation between increasing **ToUsePercent** values and the correlation values showcased in Table 9. We can see that for almost all satellite constellations, the ‘SwapWithToUse’ algorithm is the best choice.

### 4.3. Memory Saturation

In our research, after demonstrating the effectiveness of our support algorithm on both static and dynamic networks, we demonstrate its usefulness in reducing memory saturation. For this, we analysed both static and dynamic networks at high entanglement generation rates.

**Figure 19:**
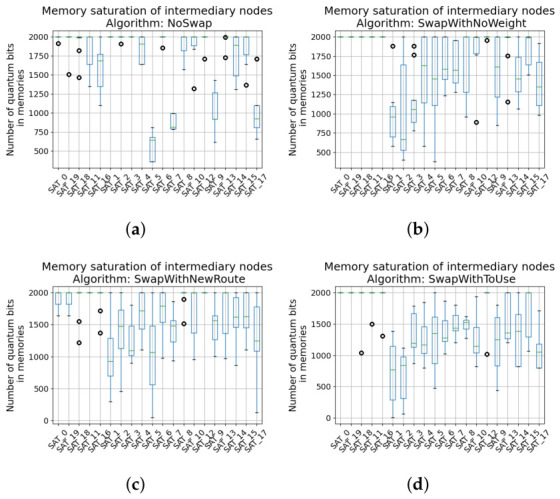
Boxplot showing memory saturation of the intermediary nodes of the Connected Watts–Strogatz graph. (**a**) Memory saturation when using the ‘NoSwap’ algorithm. (**b**) Memory saturation when using the ‘SwapWithNoWeight’ swap algorithm. (**c**) Memory saturation when using the ‘SwapWithNewRoute’ swap algorithm. (**d**) Memory saturation when using the ‘SwapWithToUse’ swap algorithm.

In Figure 19, we can see the boxplot of per-node memory saturation of the Connected Watts–Strogatz graph. It is evident that utilising our novel support algorithms enable reducing the memory saturation of the static network.

**Figure 20:**
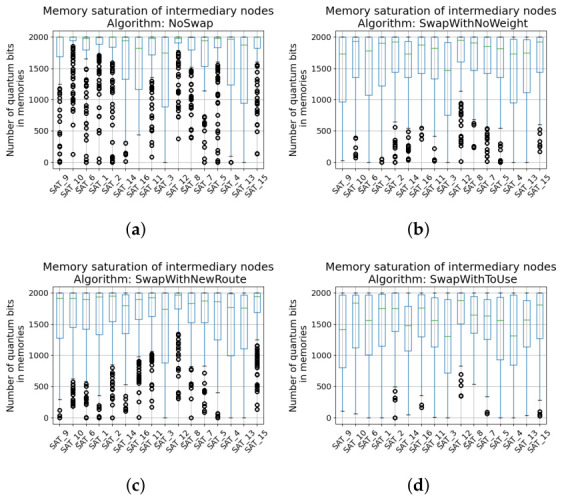
Boxplot showing memory saturation of the intermediary (satellite) nodes of the Cross LOW constellation. (**a**) Memory saturation when using the ‘NoSwap’ algorithm. (**b**) Memory saturation when using the ‘SwapWithNoWeight’ swap algorithm. (**c**) Memory saturation when using the ‘SwapWithNewRoute’ swap algorithm. (**d**) Memory saturation when using the ‘SwapWithToUse’ swap algorithm.

We show in Figure 20 the boxplot of memory saturation of the intermediary (satellite) nodes of our Cross LOW constellation. In the figure, we see that our novel support algorithms can reduce memory saturation in a dynamic network. We can see that the best-performing algorithm (SwapWithToUse) in terms of added benefit also has the lowest level of memory saturation in the context of both static and dynamic networks.

### 4.4. Comparison Against Other Routing Algorithms

After showing the usefulness of our algorithm, we wanted to compare it to other existing routing solutions. We implemented an entanglement-aware (wide-path) baseline [34], and a memory-aware (congestion-aware) baseline [35]. We wanted to see if our support algorithm could benefit even existing solutions, so for each comparison routing algorithm, we also applied our best-performing support routing algorithm (SwapWithToUse). In Table 11, we show the performance of the three routing algorithms across the four graph architectures. We can see that, as expected, in almost all cases, one of the two new routing algorithms outperforms our initial crude round robin routing. What is more interesting is that, by utilising the new primary routing algorithms, we can achieve an even higher total shared entanglement value with the ‘SwapWithToUse’ algorithm.

In Table 12, we show the comparison of different primary routing strategies on multiple satellite constellations. It is interesting to note that in all cases, the inclusion of the support algorithm provided an increase in total shared entanglements.

We have shown that our support routing algorithm can safely integrate with almost any routing algorithm and, in most cases, can even provide a higher level of improvement than the original round robin routing.

## 5. Conclusions

In this paper, we studied saturation of quantum memories caused by higher entanglement generation rates and presented a novel algorithm to address this issue. Our algorithm differs from existing solutions by using ’leftover’ entanglements after routing takes effect, without accounting for current entanglement requests across the network. This allows our algorithm to be independent of the location and capacity of end nodes. Because the selected swaps share no entanglement, the resulting changes remain local and mutually independent, allowing the algorithm to be layered on top of any existing routing scheme without network-wide coordination and independent of the location or capacity of the end nodes.

Across the static fibre topologies, the benefit of our support algorithm correlated with network connectivity. In the well-connected, connected Watts–Strogatz and Hypercube topologies, the ‘SwapWithToUse’ variant produced the largest gains, a 23-fold increase in total shared entanglements for Watts–Strogatz, and a 54-fold increase in mean for Hypercube topology. The scale-free Barabási–Albert topology showed more modest but consistent improvements (1.72 total, 1.14 mean), which we attribute to its hub-dominated structure, which limits the number of independent swap pairs the algorithm can identify. In contrast, the random geometric topology saw little to no benefit, with mean ratios below one for all three variants: in a poorly interconnected, partially disconnected network, the entanglements consumed by the initial routing algorithm are no longer available for our algorithm, so the supplementary layer competes with rather than complements the base scheme. This connectivity dependence is a central finding—the algorithm helps most precisely where there is spare, well-connected capacity for it to exploit.

In dynamic satellite topologies, we observed steady improvement for both constellation types. For both Cross and Retro constellations, as the network size increased, the improvement ratio slowly decreased. This is due to the asymmetric nature of satellite links: satellite-to-satellite links incur lower loss. In contrast, the most critical satellite-to-ground links incur the highest losses, limiting the overall network performance. When compared, our support algorithm led to greater improvements for the Cross architecture, with higher correlations between benefits and entanglement generation. The Retro architecture had a higher initial level of total and mean shared entanglements, leading to lower improvements due to the bottleneck effect of satellite-to-ground links.

For both static and dynamic networks, our novel algorithm achieved substantial improvements in almost all cases. The cause of the improvement is twofold: by utilising entanglements that are not “needed” by the primary routing algorithm, the support algorithm decreases memory saturation for both static and dynamic networks. The support algorithm also increases network connectivity, creating a smaller, more well-connected graph. When comparing to other routing methods, we saw that even if they were memory- or entanglement-aware, they still benefited from using our algorithm in most cases.

In this work, we have shown that using a support algorithm can lead to significant improvements in network performance. We believe that these algorithms are not meant to replace or limit existing well-made routing algorithms, but to enhance and support them. Including these algorithms in future networks will play a key role in building the quantum internet.

Although our proposed algorithm improved the network’s performance in our simulations, this field still requires further in-depth research, particularly in two directions: in future work, we plan to assess the usability of our algorithm for static networks from a more analytical perspective, examining where it starts to become more efficient in terms of different random graph parameters. We would also like to explore the use of our algorithm for dynamic, primarily satellite-based quantum networks from a satellite constellation planning perspective.

## Figures and Tables

**Table 1:** Key simulation parameters used in all simulations with short descriptions.

Parameter Name	Parameter Values	Short Description
QMemSize	2000	The number of quantum memory available for each node
MaxEntToLeave	200–800	The number of entanglements to leave after performing entanglement swaps
ToUsePercent	0.3–0.7	Percentage of available entanglements to use by our algorithm
EntGenRate	100–1900	The base rate of entanglement generation for the network

**Table 2:** Algorithm performance ratios relative to ‘NoSwap’ for static network architectures, averaged over entanglement generation rates 1000–1900 at an entanglement usage ratio of 0.7.

Architecture	Total Ratio			Mean Ratio		
	NoWeight	NewRoute	ToUse	NoWeight	NewRoute	ToUse
BaAl	1.1802	1.0223	1.7207	1.1162	1.0439	1.1350
HYPER	5.9748	3.6232	14.7403	26.1132	16.9871	54.0588
RGEO	0.9279	0.9235	1.1999	0.7658	0.6945	0.9362
WaSt	8.9420	2.8014	23.0749	24.5503	8.0693	42.8961

**Table 3:** Pearson correlation between the network’s entanglement generation rate and the benefit of using our algorithm. We calculated the benefit by subtracting the total entanglement generation of the network with the ‘NoSwap’ algorithm from each algorithm’s total entanglement generation. The highest value for each row is highlighted in bold. Entanglement reserve: 200. Entanglement percentage to use: 0.6.

	SwapWithNewRoute	SwapWithNoWeight	SwapWithToUse
WaSt	−0.76	−0.72	**−0.25**
BaAl	−0.48	−0.37	**0.70**
HYPER	−0.44	−0.56	**−0.01**
RGEO	−0.73	−0.87	**−0.36**

**Table 4:** Pearson correlation between the benefit correlation (correlation between benefit and entanglement generation rate) and the value of the entanglement percentage to use. The highest value for each row is highlighted in bold. Entanglement reserve: 200.

	SwapWithNewRoute	SwapWithNoWeight	SwapWithToUse
BaAl	0.72	**0.99**	0.98
HYPER	0.65	−0.06	**0.95**
RGEO	**0.99**	0.75	0.80
WaSt	0.24	0.99	**0.99**

**Table 5:** Satellite orbits used in simulations.

Constellation	LOW	LOWMID	MID
Number of satellites	16	32	64
Apogee	1000 [km]		
Inclination	56.0568°		
Eccentricity	0.0002090		
Argument of periapsis interval	0–180°		
Ascending node interval	0–180°		
Inclination shift	90° for Cross and 180° for Retro architecture		

**Table 6:** Parameters used to calculate transmittance.

Parameter Name	Parameter Type	Value	Explanation
Duration	Static	2 × 3600	Simulation length in seconds
Detector efficiency	Static	0.7	The efficiency of photon detector
Weather	Static	Clear	Weather around the ground station
Season	Static	Summer	The season around the ground station
Wavelength used	Static	860 [nm]	The wavelength of photons used in the communication
Aperture diameter	Static	0.2 [m]	The size of the aperture
Mirror diameter	Static	2 [m]	The size of the mirror
Zenith angle	Dynamic	[degree]	The angle at which a ground station can see the satellite
Distance	Dynamic	[m]	The distance between communication parties

**Table 7:** Algorithm performance ratios relative to ‘NoSwap’ for Cross constellation networks, averaged over entanglement generation rates 1300–1900 at an entanglement usage ratio of 0.7.

Architecture	Total Ratio			Mean Ratio		
	NoWeight	NewRoute	ToUse	NoWeight	NewRoute	ToUse
Cross LOW	2.0995	1.8990	2.4528	1.8332	1.6921	2.0634
Cross LOWMID	1.5570	1.4578	1.7469	1.5424	1.4316	1.6061
Cross MID	1.3021	1.2266	1.4966	1.3956	1.3253	1.5452

**Table 8:** Algorithm performance ratios relative to ‘NoSwap’ for retro constellation networks, averaged over entanglement generation rates 1000–1900 at an entanglement usage ratio of 0.7.

Architecture	Total Ratio			Mean Ratio		
	NoWeight	NewRoute	ToUse	NoWeight	NewRoute	ToUse
Retro LOW	1.9187	1.8604	2.1728	1.9358	1.9111	2.1478
Retro LOWMID	1.3901	1.3677	1.6092	1.5031	1.4674	1.7097
Retro MID	1.3202	1.2167	1.4740	1.2711	1.2060	1.3587

**Table 9:** Pearson correlation between the network’s entanglement generation rate and the benefit of using our algorithm. We calculated the benefit by subtracting the total entanglement generation of the network with the ‘NoSwap’ algorithm from each algorithm’s total entanglement generation. The highest value for each row is highlighted in bold. Entanglement reserve: 200. Entanglement percentage to use: 0.7.

	‘SwapWithNewRoute’	‘SwapWithNoWeight’	‘SwapWithToUse’
Cross LOW	0.93	0.92	**0.98**
Retro LOW	0.91	0.93	**0.97**
Cross LOWMID	0.85	0.95	**0.98**
Retro LOWMID	0.93	0.93	**0.95**
Cross MID	0.86	0.89	**0.98**
Retro MID	0.80	0.87	**0.96**

**Table 10:** Pearson correlation between the benefit correlation (correlation between benefit and entanglement generation rate) and the value of the entanglement percentage to use. The highest value for each row is highlighted in bold. Entanglement reserve: 200.

	‘SwapWithNewRoute’	‘SwapWithNoWeight’	‘SwapWithToUse’
Cross LOW	0.47	−0.59	**0.74**
Cross LOWMID	0.61	0.55	**0.82**
Cross MID	**0.78**	−0.31	0.45
Retro LOW	−0.26	0.21	**0.76**
Retro LOWMID	**0.86**	0.36	0.12
Retro MID	0.03	**0.23**	−0.50

**Table 11:** Comparison table for static architectures, showing total shared entanglements across the network for three routing algorithms (round robin, entanglement aware and memory aware). For each architecture and primary routing combination, we highlight the higher value with bolding. Entanglement generation rate: 1800; ToUsePercent: 0.7; MaxEntToLeave: 200.

Architecture	Routing	‘NoSwap’	‘SwapWithToUse’
BaAl	Entanglement aware	**865,948.0**	788,784.0
	Memory aware	577,572.0	**821,966.0**
	Round robin	**788,079.0**	774,420.0
HYPER	Entanglement aware	84,860.0	**567,403.0**
	Memory aware	277,535.0	**448,506.0**
	Round robin	66,813.0	**455,225.0**
RGEO	Entanglement aware	339,633.0	**738,958.0**
	Memory aware	445,366.0	**796,456.0**
	Round robin	445,530.0	**622,072.0**
WaSt	Entanglement aware	318,536.0	**630,382.0**
	Memory aware	131,972.0	**626,665.0**
	Round robin	310,929.0	**575,158.0**

**Table 12:** Comparison table for satellite architectures, showing total shared entanglements across the network for three routing algorithms (round robin, entanglement aware and memory aware). For each architecture and primary routing combination, we highlight the higher value with bolding. Entanglement generation rate: 1800; ToUsePercent: 0.7; MaxEntToLeave: 200.

Architecture	Routing	‘NoSwap’	‘SwapWithToUse’
Cross LOW	Entanglement aware	628,536.0	**1,416,030.0**
	Memory aware	722,023.0	**1,631,874.0**
	Round robin	541,174.0	**1,630,334.0**
Cross LOWMID	Entanglement aware	1,712,837.0	**3,020,345.0**
	Memory aware	1,614,982.0	**2,960,491.0**
	Round robin	1,802,433.0	**3,049,361.0**
Cross MID	Entanglement aware	3,931,719.0	**5,520,633.0**
	Memory aware	4,035,062.0	**6,242,631.0**
	Round robin	4,023,761.0	**6,128,382.0**
Retro LOW	Entanglement aware	918,415.0	**1,881,479.0**
	Memory aware	955,599.0	**1,920,227.0**
	Round robin	860,991.0	**2,072,627.0**
Retro LOWMID	Entanglement aware	3,388,666.0	**4,781,074.0**
	Memory aware	2,944,964.0	**4,689,312.0**
	Round robin	2,817,788.0	**4,324,959.0**
Retro MID	Entanglement aware	4,176,816.0	**5,957,967.0**
	Memory aware	4,166,170.0	**5,904,483.0**
	Round robin	4,171,598.0	**6,506,282.0**

## Data Availability

Data available on request to the corresponding author.
